# Clinically Undiagnosed Prostate Carcinoma Metastatic to Renal Oncocytoma

**DOI:** 10.1155/2012/307813

**Published:** 2012-06-13

**Authors:** Adam J. Horn, Bari E. Fritz, Chad A. LaGrange, William W. West, Subodh M. Lele

**Affiliations:** ^1^Department of Pathology and Microbiology, University of Nebraska Medical Center, Omaha, NE 68198, USA; ^2^Division of Urology, Department of Surgery, University of Nebraska Medical Center, Omaha, NE 68198, USA

## Abstract

Tumors-to-tumor metastasis is an uncommon occurrence and can be a source of great diagnostic difficulty, especially when the donor tumor is undiagnosed. Here we report a case of a kidney resected for a primary neoplasm (oncocytoma) that harbored metastases from a clinically undiagnosed prostatic adenocarcinoma. The presence of the poorly differentiated metastasis within an otherwise typical oncocytoma in the absence of metastases in the surrounding nonneoplastic renal parenchyma resulted in a diagnostic dilemma. To our knowledge, this is the first report of a case in the English literature of a clinically undiagnosed prostatic adenocarcinoma metastatic to a renal oncocytoma identified on examination of the resected renal neoplasm.

## 1. Introduction

While tumor metastasis is a common occurrence, the tumor-to-tumor metastasis is quite rare, being first documented in 1902. The most common donor site is lung, followed by breast, prostate, and thyroid carcinomas [1]. The kidney (in particular, renal cell carcinoma) is the most common recipient, followed by sarcomas, meningiomas, thyroid, and pituitary adenomas [1, 2]. In addition to renal cell carcinoma, angiomyolipomas and renal oncocytomas have been described as being recipients [3, 4]. In this paper we present a case of renal oncocytoma harboring metastases from a previously undiagnosed prostatic adenocarcinoma. To the best of our knowledge, there has been no documented cases of prostatic adenocarcinoma with metastasis to a renal oncocytoma.

## 2. Case Report

A 92-year-old man presented with complaints of low back pain. He had magnetic resonance imaging of the spine performed which incidentally revealed a mass in the left kidney. A CT scan confirmed this finding, demonstrating a 6 cm solid enhancing mass in the left kidney, in addition to multiple bilateral lung nodules and lesions in the spine worrisome for metastatic disease. He was presumed to have metastatic renal cell carcinoma and underwent a radical nephrectomy.

**Figure 1:**
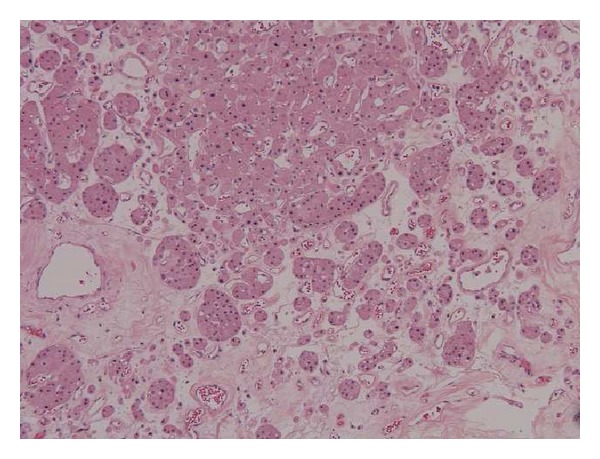
Oncocytoma. Note the archipelaginous architectural pattern with oncocytic cells (hematoxylin and eosin; original magnification × 40).

**Figure 2:**
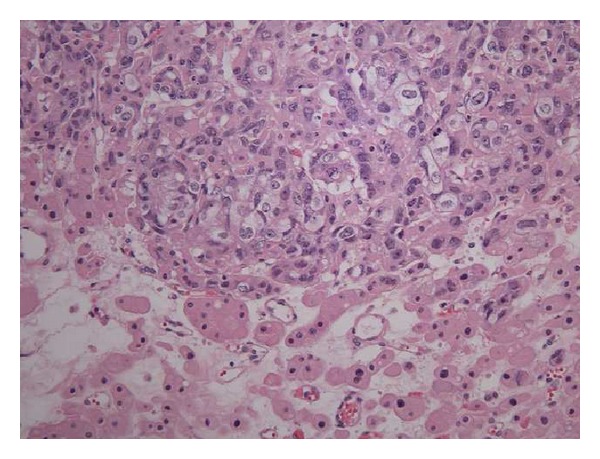
A second population of cells with malignant cytologic features is noted admixed within the oncocytoma (hematoxylin and eosin; original magnification × 200).

Dissection of the radical nephrectomy specimen revealed a solitary tumor measuring 7.4 × 5.8 × 3.5 cm, which was well circumscribed, tan-brown, and confined to the kidney. Microscopic examination of the renal tumor revealed areas typical of an oncocytoma with an archipelaginous architectural pattern near the center of the tumor (Figure 1). In addition to the oncocytic cells, a second population of cells arranged in small groups containing high nuclear/cytoplasmic ratios, nuclear hyperchromasia, occasional prominent nucleoli, and rare mitoses were noted (Figure 2). The nests were restricted to the oncocytic neoplasm and were not present in several additional sections from the surrounding grossly unremarkable renal parenchyma. The second population of cells was strongly positive for PSA, PAP, cytokeratin (AE1/AE3), and EMA, confirming the diagnosis of prostatic adenocarcinoma metastatic to renal oncocytoma. The pathologic diagnosis led to testing for serum PSA (>500 ng/mL), and the subsequent prostate needle biopsy demonstrated a high-grade prostatic adenocarcinoma with a Gleason score of 9 (4 + 5) that was morphologically similar to the metastatic deposits within the renal oncocytoma.

## 3. Discussion

Although the incidence of more than one neoplastic processe occurring simultaneously in a patient ranges from 4.2 to 8%, the documented metastasis of one neoplasm to another is extremely rare, with less than 100 cases being reported in the English literature [3, 5, 6]. It is theorized that neoplasms produce substances that restrict the growth of other cells [1, 7]. It has also been suggested that the rapid growth of neoplasms creates a nutritionally competitive environment [8, 9]. Campbell et al. described criteria for the diagnosis of a tumor-to-tumor metastasis: (1) more than 1 primary tumor must exist, (2) the recipient tumor is a true benign or malignant neoplasm, (3) the metastatic neoplasm is a true metastasis with established growth in the host tumor, not the result of contiguous growth (a so-called “collision tumor”) or tumor embolization, and (4) neoplasms that have metastasized to the lymphatic system where lymphoreticular tumors already exist are excluded. This case fits all criteria.

Prostatic adenocarcinoma has high propensity for metastasis to bone and much metastasizes to visceral organs with far less frequency. One explanation for the strong attraction to bone is that prostate tumor cells will bind to human bone marrow endothelial cells with a higher affinity than other endothelial cells [10]. In cases of tumor-to-tumor metastasis, prostate cancer is a rare donor tumor, with 4 previously reported cases metastasizing to renal cell carcinoma, 1 to a follicular adenoma of the thyroid, and 1 to a pituitary adenoma [8, 11–15].

The kidney is a common recipient for tumor-to-tumor metastasis, which is thought to be partially due to the rich vascular supply of the kidney [1, 6]. Renal cell carcinoma is the most common recipient, with over 60 cases being reported [1]. However, there has only been 3 previously described cases of metastatic disease to renal oncocytoma (Table 1) [3, 16, 17]. This case represents the first reported case of prostatic adenocarcinoma to renal oncocytoma. This case is also of interest because the prostate cancer was undiagnosed at the time of its discovery within the oncocytoma.

In conclusion, this case emphasizes the need to entertain tumor-to-tumor metastasis when encountering a diagnostically difficult or unusual case. The judicious use of immunoperoxidase staining panels can be of assistance in reaching a final, accurate diagnosis.

## Figures and Tables

**Table 1:** Known cases of tumor-to-tumor metastasis with renal oncocytoma as recipient.

	Age	Sex	Donor Site
Olsen et al. [17]	71	Male	Small cell, lung
Ben-Izhak et al. [16]	63	Male	Small cell, lung
Altinok et al. [3]	64	Male	Squamous cell carcinoma, lung
Present case	92	Male	Prostatic adenocarcinoma
